# Dysphoric milk ejection reflex: A case report

**DOI:** 10.1186/1746-4358-6-6

**Published:** 2011-06-06

**Authors:** Alia M Heise, Diane Wiessinger

**Affiliations:** 1 Finger Lakes WIC, PO Box 521, Naples, NY 14512, USA; 2 136 Ellis Hollow Creek Road, Ithaca, NY 14850, USA

## Abstract

Dysphoric Milk Ejection Reflex (D-MER) is an abrupt emotional "drop" that occurs in some women just before milk release and continues for not more than a few minutes. The brief negative feelings range in severity from wistfulness to self-loathing, and appear to have a physiological cause. The authors suggest that an abrupt drop in dopamine may occur when milk release is triggered, resulting in a real or relative brief dopamine deficit for affected women. Clinicians can support women with D-MER in several ways; often, simply knowing that it is a recognized phenomenon makes the condition tolerable. Further study is needed.

## Background

Because successful milk feeding is vital to mammalian newborn survival, it is essential that the mother be drawn to provide it, and that it feel both comfortable and desirable when milk flows efficiently and effectively. Oxytocin, which is central to milk release, is described by Uvnäs Moberg as a hormone of calm and connection [1].

However, there is a subset of human mothers for whom every breastfeeding session includes periods of negative feelings. Named "Dysphoric Milk Ejection Reflex" or "D-MER" by Heise, co-author, it is a breastfeeding problem for which affected mothers seem only rarely to seek or receive help.

Since D-MER is only recently recognized, literature is limited and direct research is non-existent. However, a range of publications indicate a growing awareness of D-MER among breastfeeding mothers and their helpers at various levels. Heise has written articles or been interviewed for several lay publications [2-4]. Cox's 2010 case study describes an Australian mother's experience with D-MER [5]. D-MER is also referred to in the most recent editions of both a professional text [6] and a popular breastfeeding book [7], and various breastfeeding websites and blogs now discuss ways of recognizing and coping with the problem.

Nonetheless, D-MER probably results in unwanted cessation of breastfeeding at times. For all affected mothers, it is a troubling, lonely experience. Support from health care providers and contact with other "D-MER mothers" can help until causes and solutions are better defined. In this paper, the authors follow the course of D-MER in one mother, present the potential solutions she tried, and propose a hormonal cause for its symptoms, based on the outcomes of those trials.

## Case presentation

AH, a breastfeeding counselor for the United States Department of Agriculture WIC (Women, Infants, and Children) Program, had had two previous unremarkable pregnancy and lactation experiences. Her third child was born at home, with no interventions and a smooth transition to the breast. Within two weeks, however, AH wondered if she might be suffering from postpartum depression for the first time; the rosy glow of the early days was interjected with periods of extreme unhappiness. She soon realized that her symptoms occurred only when her milk was about to release, continuing for perhaps ninety seconds to two minutes, ending as quickly as they began, but recurring with the next milk release. Certain that her problem was hormonal, she could neither find information about it nor "talk herself out of it." Between milk releases, she felt extremely happy and well-bonded to her baby. Just before each milk release, however, and despite her best efforts, she felt helpless, hopeless, worthless. Her milk supply was excellent, tending toward oversupply as it had with her first but not her second child.

Direct nipple stimulation was not needed for the onset of negative emotions. Anything that caused a milk release, expected or unexpected - breastfeeding, mechanical or manual milk expression, the release of milk caused by thinking about her baby or by breast fullness - generated the same negative feelings. AH experienced very frequent spontaneous (no nipple stimulation) milk releases during the early months. If she was shopping when a spontaneous milk release occurred, she suddenly felt that she was choosing ugly clothes. If she was cooking, she knew instantly that she was preparing a meal that her family would hate. If she was doing nothing at all when one occurred, she immediately believed herself to be worthless or worse. During her daughter's later infancy, she averaged perhaps five spontaneous milk releases per day; by 18 months to two years, she experienced one to two per day. Each one of these involved a D-MER. They were often worse than those that occurred during breastfeeding, perhaps because at those times she was caught unaware and there was no baby to offer a distraction.

In addition to the invariable emotional sinking that accompanied D-MER, AH's ability to concentrate faltered with each episode. Simple math was temporarily impossible; she experimented with mentally reciting the times tables as a distraction, and stalled at 2 × 3. AH was given a brief, affirming statement to read to herself during a D-MER. She could neither read it easily, nor believe it at all, until the feelings passed.

AH also felt a physical undertone with milk release, in the form of a hollowness in the pit of her stomach, and while she did not become nauseated, food and drink felt abruptly and briefly disgusting. However, her primary feelings were emotional: she was overwhelmed by feelings of low self-worth, guilt, hopelessness, shame, and a desire to hide from the world. The emotions were directed toward herself, not toward her child; her bond with this child, following such a good birth, was her strongest yet. However, she anticipated feedings not with pleasure or indifference, but with a certain dread.

When her baby was seven months old, AH e-mailed DW, co-author, who lives in another city. DW's e-mail response, like those of several International Board Certified Lactation Consultants (IBCLCs) and physicians before her, was nothing more than general reassurance and a suggestion that postpartum depression might be involved. Two months later, AH tried again, this time by phone. This time, DW listened. She learned that AH's D-MER, unlike depression, could quickly be improved, worsened, or even briefly eliminated by very specific environmental or chemical changes. Over time, AH and DW were able to add activities to the list (Table 1), a key part of their search for a cause and thus a solution.

**Table 1:** Activities that seemed to affect D-MER, with their presumed effects on dopamine and oxytocin

Activity	Dopamine	Oxytocin	Observed effect on D-MER symptoms	Comments
Alcohol - one serving	**0**	**0 **[24]	none	

Smoking - two to five cigarettes in rapid succession	↑	↓ [25]	improved	Numerous studies show an increase in dopamine with exposure to nicotine.

Pseudo-ephedrine (two 30 mg tablets)	↑ [10]	**0?**	improved	PSE *may *reduce milk supply by reducing prolactin, implying an increase in dopamine. No literature found on the effect of pseudoephedrine on oxytocin release.

Bupropion - 150 mg/day	↑ [21]	**0?**	improved	No literature found on the effect of bupropion on oxytocin release.

Chocolate ice cream binge	↑ [1,26]	↑	improved	Occasional evening binges were followed by a small window of D-MER-free breastfeeding.

Chronic moderate stress	↑	↑	improved	

Caffeine	↑and↓	**0**	worsened	Some internet hints of a dopamine rise and subsequent "crash" were not verified

Acute stress	↓ [27]	↑ [28]	worsened	

Metoclopramide	↓ [29]	**0**	worsened	Administered by IV during pregnancy; reaction was similar to D-MER

Immediately after meals with extended family	**?**	**0**	worsened	Relations with extended family were good. While the worsening of symptoms was noticeable at family gatherings, we have no explanation.

Note: 0 = neurotransmitter release unaffected by activity/drug listed; ↑ = neurotransmitter release increased by that activity/drug; ↓ = neurotransmitter release decreased; ? = uncertainty about effect of activity/drug on neurotransmitter

### The D-MER website

When AH realized that her dysphoria and her milk releases were connected, the first place she looked for information was the internet. But there was no information to find, other than a few short threads on messages boards started by women brave enough to talk about it. When her daughter was 11 months old, in June 2008, AH established the website http://d-mer.org[8], and created a questionnaire, both to find and connect fellow sufferers, and to find solutions. During its first several months, over 100 women responded with D-MER stories of their own.

At this writing, the site has had over 30,000 hits, both from the United States and internationally. Hundreds of women had indicated that they have, or have had, experiences similar to AH's. The website now includes first-person accounts by other D-MER mothers, handouts that mothers can share with their health care providers, and networking opportunities.

The connection with others through the website has been a powerful experience for both AH and the respondents:

"I thought I was just a bad mom, maybe I wasn't cut out for mothering, and that wasn't something that I really wanted to tell anyone."

"Until now I never realized anyone else had this issue - I would feel profoundly depressed at time of letdown and my doctor and pediatrician both dismissed my complaints."

None of the mothers believed it to be a purely psychological problem, although some had worried before finding the site that something in their past or present might have contributed:

"I wondered if there was abuse or some other negative experience I was repressing."

For the average sufferer of D-MER, the reassurance that it is almost certainly physiological may be enough:

"My feelings during D-MER could best be described as self-disgust and hopelessness. I don't feel my case is especially severe and for the most part, understanding that these feelings are just the D-MER and dismissing them (vs. dwelling on them) has been sufficient for me."

"Just knowing it is not dangerous is enough."

### The experience and spectrum of D-MER

There is a recognized link between some hormonal shifts and dysphoria; information on "premenstrual dysphoric disorder" was well summarized by Braverman in 2007 [9]. However, D-MER is quite different in its onset and duration.

Imagine tapping your knee to cause a reflexive jerk. Now tell yourself that you are going to resist the reflex with all your will, and tap again. Does your willpower make any difference? This is precisely the problem for the mother with D-MER. The emotions are unavoidable. She can feel them coming, but cannot stop them. The mother can tell herself they are not based in reality, can brace against them, can will them to stop. Some feel them with every milk release, others only with the first one in a feeding, but like a reflex, the emotions recur unavoidably with the next feeding. Now imagine that you stop tapping your knee. Does it continue to jerk? In the same way, once a D-MER passes, the dysphoria is completely gone. The thoughts and feelings that are unavoidable at one moment have disappeared in the next.

Most of the website's respondents felt dysphoria regardless of the cause of milk release, although a significant number did not feel it during pumping. Few of the mothers who have written to the website noticed their negative emotions immediately post-birth. Within two weeks of the birth, more than half were aware that something was amiss; linking it to milk release took longer:

"Without fully understanding [what was happening], it was difficult to search for an explanation. All I ever found was that breastfeeding could help with postpartum depression - and I didn't think I had postpartum depression."

Rather than forming a continuum like the numbers on a scale, D-MER symptoms seem to form a spectrum of three *distinct and different emotions *(depression, anxiety, anger) whose intensity varies from woman to woman (see Table 2). AH feels she has had "severe depressive symptoms"; although they lasted well into her baby's toddlerhood and at their worst were nearly disabling, they never included such adjectives as "anxious" or "angry". Those with mild depressive D-MER may barely notice an experience that is limited to brief feelings of wistfulness or homesickness, and usually find that it ends altogether within a few months. In general, the more intense the experience, the longer it is likely to last. And in general, mothers with "anxious" symptoms seem less likely to feel them mildly, and those with "angry" symptoms seem less likely to feel them mildly or even moderately. This may not be surprising, since anxious and angry emotions are by their nature more intense; the phrases "mildly irritable" and "somewhat angry" feel contradictory to most of us.

**Table 2:** The D-MER spectrum of symptoms and associated intensities

Symptoms	1 Depression wistfulness, homesickness, apprehension, hopelessness, hollowness in stomach	2 Anxiety anxiety, dread, panic, irritability	3 Anger tension, agitation, paranoia
Intensity of symptoms			
**Mild **usually self-corrects within first 3 months, most mothers do not seek treatment	**X**		

**Moderate **often self-corrects between 6 and 12 months, mothers may seek management options	**X**	**X**	

**Severe **may not correct fully until weaning, may include transient suicidal ideation, prescription treatment may be only effective option, mothers likely to stop breastfeeding	**X**	**X**	**X**

Approximately half of women who wrote after weaning said that their symptoms did not disappear completely until they weaned. Others found that their symptoms dissipated around the end of lactational amenorrhea or with the decrease in supply and feeding frequency that accompanies breastfeeding an older baby or toddler. It isn't clear whether all cases of D-MER resolve before natural weaning would occur; very few women with a severe case seem able to continue to breastfeed long enough to find out.

According to mothers' comments, D-MER typically seems to occur either with all children, or with later children. Perhaps D-MER is one of the many potential "glitches" that may accumulate as we age. Perhaps the increased age of today's new mothers or accumulated environmental insults have resulted in the frequency we seem to see today. It seems unlikely that something as aversive as D-MER can be has always occurred with today's apparent frequency.

Most of the website's respondents indicated that they would be happy to help with research. These women pay a considerable emotional price in order to breastfeed their children. Knowing that someone might be able to use their experience to help others could make an unhappy experience a little more bearable.

### The case for dopamine

At first, the authors assumed that oxytocin was involved, since D-MER coincides so reliably, abruptly, and uniquely with milk release. But factors affecting oxytocin levels did not always affect AH's D-MER, while other factors unrelated to oxytocin levels sometimes increased or decreased the intensity. Informal discussions with other professionals specializing in drugs, counseling, oxytocin, or neurobiology ultimately resulted in a small team of colleagues who provided suggestions, information, encouragement . . . and brainstorming, during which the word "dopamine" was first mentioned. It seems to the authors to be a very good fit (see Table 1). On one occasion, for instance, AH took pseudoephedrine for cold symptoms, unaware of its potentially negative effect on milk production. Within hours she called DW asking, "What happened? My D-MER is gone!" Pseudoephedrine does not appear to influence oxytocin, but it does slightly reduce prolactin [10]. Might it do so by increasing dopamine?

Dopamine, produced in several areas in the brain, is a hormone and neurotransmitter that holds prolactin in check. If dopamine rises, prolactin falls; if dopamine falls, prolactin rises. It is probably the major prolactin-inhibiting factor, or PIF [11], in humans, although there may be minor factors that affect prolactin indirectly by affecting dopamine. In lactating women, prolactin levels rise and fall over a period of many minutes, even hours, in a pattern very different from that of D-MER [12]. Dopamine's rise and fall during breastfeeding is less well researched, but it may be much more abrupt, reflecting the spiky pattern not only of oxytocin, but of D-MER.

Direct observation of neurotransmitter levels is extremely difficult. A brain is not nearly as easily invaded as a bloodstream, and so the precise timing and interactions of dopamine, oxytocin, and prolactin releases are unclear, especially in humans. In some studies, dopamine *added *to rats' cerebral ventricles resulted in the *release *of oxytocin [13,14]. In contrast, Seybold, *et al*. [15], found that dopamine *inhibited *oxytocin release in rats. In an unreferenced statement, Conn and Freeman [16] implicate dopamine and glutamate in the release of oxytocin, without stating whether a rise or fall in dopamine is involved. (Glutamate may be the messenger that carries environmental signals to dopaminergic cells for interpretation [17]). Knight, *et al*. [18], observed that a "transient reduction in the secretion of dopamine into the hypophyseal portal vessels" (blood vessels linking the hypothalamus to the anterior pituitary) resulted in elevated prolactin levels in ewes, without specifying exactly when or for how long that transient dopamine reduction occurred. Plotsky and Neili [19] found that mammary nerve stimulation produced "a momentary, but profound, decrease in hypothalamic dopamine secretion . . ." "Momentary but profound" certainly fits the D-MER experience, though whether the timing of the decrease coincides roughly with the timing of oxytocin release is not discussed.

Faure, *et al. *[17], and Berridge [quoted in [20]] describe a "keyboard" of dopamine-mediated emotions, ranging from desire to disgust, triggered by the amino acid glutamate acting on varying locations of the brain's nucleus accumbens. Depending on precisely where they disrupt glutamate reception, their team is able to create either fear or pleasure-based binge-eating in their rat subjects. They suggest that some people may have a skewing of their dopamine "keys", resulting in emotions not typical for the stimuli received. Some D-MER mothers have volunteered the word "homesick" to describe their emotion - a surprising word for more than one woman to offer spontaneously, and one that could be considered the unhappy remembering of a happy time. Might homesickness, wistfulness, and longing - all words used by women with depressive-type D-MER - represent the balance point between pleasure and pain on the dopamine "keyboard"? Might the three categories of D-MER symptoms reflect a skewing of different parts of the keyboard? And, most exciting of all, might D-MER mothers be of use to researchers because they are able to describe their emotional "keyboard" so precisely, providing a non-invasive window onto one neurotransmitter's activity?

Anecdotally, two women reported an abrupt and transient dysphoria with perimenopausal hot flashes. Sexually-based nipple play, dubbed "sad nipple syndrome" in an on-line discussion, may also create a brief dysphoria in some women. Transient emotional skewing may not be limited to women. One woman's husband wrote:

"For much of my life - I'm now 65 - I've experienced infrequent, sudden, unexplained dips in my emotions while I'm thinking about something positive. I'll be having pleasant thoughts about an upcoming trip, for instance, only to have them suddenly and almost forcibly replaced with bad feelings, and thoughts of lost tickets, missed connections, a bad time. These 'down' feelings never last very long - a minute or less - and I quickly rebound to the pleasant thoughts."

Perhaps the experience of abrupt and transient dysphoria is fairly common in both men and women. What makes the D-MER mothers potentially valuable to researchers is the ease and predictability of creating the dip: trigger a milk release, and an emotional dip is triggered.

Based on AH's experiences, and until more is known, it is the authors' very humble hypothesis that a drop in dopamine either facilitates or parallels each oxytocin spike in lactating human mothers, and that it is this dopamine drop that results in D-MER in susceptible mothers. Other ideas that were considered - nutritional or blood glucose deficiencies, for instance - did not explain the outcomes in Table 1 as well as a drop in dopamine did.

The "low dopamine" hypothesis helped AH find a solution for herself, which strengthened the authors' suspicion that dopamine was involved. A "reuptake inhibitor" is a drug that increases the availability of a particular neurotransmitter, effectively increasing its level. A minimal dose of bupropion, a dopamine reuptake inhibitor [21], reduced AH's D-MER within a day and virtually eliminated it within five days. Unfortunately, although bupropion did not alter her milk supply significantly, it had other side effects that prevented her from continuing to take it beyond a few weeks; D-MER promptly returned.

By the time her daughter was about 14 months old, AH's D-MER symptoms were somewhat reduced, but still extremely troublesome. She learned that *Rhodiola rosea*, also called Arctic root or golden root [22], is a monoamine oxidase inhibitor that prevents the breakdown of dopamine, increasing its availability. While herbal remedies do not have the safety oversight of prescription drugs, Humphrey [23] gives Rhodiola a safety rating of B: "May not be appropriate for self-use by some individuals or dyads, or may cause adverse effects if misused. Seek reliable safety and dose information." Humphrey groups it with several other "tonic herbs" for which "there are no anecdotes, as yet, to suggest that they will affect breastfeeding babies." Any herbal remedies should of course be used with caution, but with the use of *Rhodiola *capsules, AH's symptoms became much milder; at times she could release milk without noticing that she had done so. No other effects or changes in milk supply were noted.

On days when AH failed to take her accustomed amount of *Rhodiola*, her D-MER symptoms were worse, indicating that it was indeed the herb that was providing relief. Gradually, however, the nuisance of remembering to take a capsule three to four times each day exceeded the unpleasantness of D-MER. As her daughter passed a year and a half and was breastfeeding less often, there were also many fewer spontaneous milk releases. With fewer episodes to cope with each day, and with their intensity lessening, D-MER became tolerable without treatment. Though it increased during the week before her periods, at its worst it simply made her "so tired she could cry." It was entirely gone by the time her nursling was three.

## Conclusions

D-MER appears to be much more common than Sheehan's Syndrome or galactosemia, with which most lactation consultants feel they need a passing familiarity. It therefore deserves a place in the lactation consultant's and health care provider's consciousness.

The signature symptom for D-MER is dysphoria. However, there is considerable evidence that it is physiological, not psychological, in nature:

• D-MER sufferers feel the dysphoria *only *during milk releases.

• For most sufferers, the dysphoria does not require breast or nipple contact, only the presence of a MER.

• D-MER seems to be independent of a woman's birth or living experiences, and can occur abruptly and intensely after several normal lactations.

• It seems to be influenced, often within minutes or hours, by circumstances that influence dopamine levels.

An open-ended question like "How do you feel during breastfeeding?" could open the door for discussion with a mother and for reassurance and guidance if needed. A proactive approach may be important because mothers may fail to report D-MER symptoms to their caregivers. Their reasons may include 1) their sense that it is not psychological, 2) their belief that anyone they tell will automatically urge counseling or antidepressants as a solution, 3) embarrassment at not having the positive feelings they were "supposed" to have, and 4) their fear that no one else has this odd reaction to breastfeeding. Alerting new mothers to the existence of D-MER allows sufferers - known and unknown - to connect with other D-MER mothers and to feel more at ease with their unusual reaction to breastfeeding. Modest comfort measures such as distraction, music, or aromatherapy may also help. Only about five percent of respondents so far have expressed interest in pursuing a prescription option, though it is helpful to know that such options may exist.

D-MER mothers have the same emotional peaks and valleys that every mother has. They recognize that this is somehow different. These mothers serve as a reminder to us all that we do not yet have a complete understanding of human lactation.

"If you read Harry Potter they talk about the creatures that suck the soul out of you and when they are around it makes you cold and you start to focus on negative things and fall into this abyss of negative thoughts - that is how D-MER was for me at times. I hope the research you are doing helps you come to some conclusions. Please let me know if there is anything else I can do."

## Consent

AH served as both research subject and co-author; this paper serves as her informed consent for publication of this case report. A copy of a written consent is available for review by the Editor-in-Chief of this journal.

## Competing interests

AH is the webmaster for http://d-mer.org. DW has no competing interests.

## Authors' contributions

AH was the primary provider of data, contacts with "D-MER mothers," and insights into D-MER's variations and consequences; DW was the primary source of professional contacts and writing. Each considers herself extremely fortunate to have found the other. Both authors have read and approved the final manuscript.
